# Exosomal microRNAs in Different Disease Activity Status in Systemic Lupus Erythematosus: A Retrospective Study

**DOI:** 10.5152/ArchRheumatol.2025.11027

**Published:** 2025-09-01

**Authors:** Wenyu Xu, Peiying Nie, Qian Li, Bingjie Gu, Qijie Ren, Xingguo Chen

**Affiliations:** 1 Department of Rheumatology and Immunology, Nanjing First Hospital, Nanjing Medical University, Nanjing, China; 2 Department of Endocrinology and Rheumatology, Shanghai University of Medicine and Health Sciences Affiliated Zhoupu Hospital, Shanghai, China

**Keywords:** Exosomes, gene chip, microRNA, systemic lupus erythematosus

## Abstract

**Background/Aims:**

Differential miRNA expression profiles in the plasma exosomes of systemic lupus erythematosus (SLE) patients were obtained at various disease activity stages and compared with healthy controls.

**Materials and Methods:**

Plasma samples were collected from 48 SLE patients with high, medium, and low disease activity and from 20 healthy controls. The sample set was retrospectively analyzed for differences in clinical features. Plasma exosomes were extracted and subjected to comprehensive detection and analysis. Total exosomal RNA was extracted from the 4 groups, and differential expression profiles were analyzed using miRNA chip technology.

**Results:**

Significant differences in clinical parameters—including platelet count (PLT), erythrocyte sedimentation rate (ESR), 24-hour urinary protein, and complement C3/C4 levels—were observed among SLE patients with different disease activity levels (all *P* < .05). Plasma exosomes were successfully isolated and characterized. Microarray analysis identified distinct exosomal miRNA profiles. Notably, let-7a-5p and miR-23a-3p were significantly downregulated in patients with high disease activity compared to those with low activity (fold change > 2, *P* < .0001), while miR-4532 was markedly upregulated. Correlation analysis showed let-7a-5p expression was positively associated with PLT (*r* = 0.61) and complement C3 (*r* = 0.69) and negatively with ESR (*r* = –0.65). Conversely, miR-4532 was positively correlated with ESR (*r* = 0.67) and urinary protein (*r* = 0.56) and negatively with C3 and C4 (both *r* = –0.67).

**Conclusion:**

Differential miRNA expression was identified in the exosomes of SLE patients at various disease activity levels. These findings indicate the crucial role of these miRNAs in the onset and progression of SLE, providing a basis for further investigation of the immunoregulatory functions of exosomes.

**Main Points**

- Exosomal miRNA profiles were analyzed in SLE patients with varying disease activity and healthy controls.
- let-7a-5p, miR-23a-3p and miR-4532 were correlated with clinical parameters.

## Introduction

Systemic lupus erythematosus is a prototypical autoimmune disease characterized by the abnormal production of autoantibodies, diverse clinical manifestations, and multi-organ damage. Fatality can occur in severe cases.^1^ Its prevalence in the Chinese population is approximately 70 per 100 000, with the disease affecting nearly 1 million individuals, primarily women of reproductive age.^2^ Despite extensive research, the knowledge of its pathogenesis remains incomplete. Current studies indicate that the pathological hallmark of SLE is the overactivation of autoreactive immune cells, leading to the continuous production of cytokines, autoantibodies, and other inflammatory factors; this induces a “cytokine storm,” which can result in immune complex deposition, multi-organ damage, and death.^3,4^

Exosomes are rich in vascular growth factor, epidermal growth factor, and non-coding RNA, which participate in cell signal transduction and influence cell biological characteristics.^5^ Exosomal microRNA (miRNA) are small molecular RNAs that regulate gene expression and play a role in target cells by regulating a variety of biological processes. Compared with miRNA in peripheral blood, exosomal miRNA is easier to detect and can more accurately reflect the expression level of real miRNA in cells.^6^

Abnormal expression of exosomal miRNAs may be a key factor in the pathogenesis of SLE-related diseases.^7^ Wu et al^8^ suggested that exosomes, which serve as intercellular messengers, can travel via the bloodstream to activate immune cells in distant locations, causing inflammation in patients with SLE. Plasma exosomes have been isolated from SLE patients and healthy controls. Polymerase chain reaction was used to detect the expression of miR-21, miR-146a, and miR-155 in the exosomes. These results suggest that the expression of miR-21 and miR-155 was upregulated in SLE patients, whereas the expression of miR-146a was downregulated. Therefore, the expression levels of miR-21 and miR-155 in exosomes may serve as potential biomarkers for the diagnosis of SLE and lupus nephritis.^9^ Studies have identified differentially expressed exosomal miRNAs and proteomes in the plasma of SLE patients and healthy controls, uncovering distinct molecules (39 miRNAs, 14 proteins) and 21 signaling pathways, suggesting their potential application in the clinical diagnosis and treatment of SLE.^10^

However, the expression of exosomal miRNA in SLE with different disease activity has not been reported. In this study, SLE patients with different disease activities were enrolled, their plasma was collected to extract exosomes, and differentially expressed miRNAs were screened using gene chip analysis. The aim was to clarify the role of exosomal miRNAs in the pathogenesis of SLE.

## Methods

### Study Population

This study was approved by the Medical Ethics Committee of Shanghai University of Medicine and Health Sciences Affiliated Zhoupu Hospital (Shanghai, China) (approval number: 2023-C-187, date: 2023-10-30), and signed informed consent was obtained from all participants. The Rheumatology Department established a specimen bank for SLE patients in January 2016. All patients included in the specimen bank fulfilled the American College of Rheumatology classification criteria (1997) for SLE.^11^ Inclusion criteria: Han Chinese background; no evidence of other immune-related diseases, cancer, or infections; Exclusion criteria: history of infection within 1 month before admission; pregnant or lactating females; other immune-related diseases; severe renal damage; presence of malignant tumors; epilepsy, peptic ulcer, active liver disease, diabetes, and other hormone application contraindications; lack of complete clinical data; and voluntarily applied for withdrawal from the study. A total of 10 mL of blood sample was collected from all patients for future retrospective studies. A total of 48 newly diagnosed patients were enrolled from the specimen bank and categorized into 3 groups based on their systemic lupus erythematosus disease activity index (SLEDAI) scores: 16 cases in the high disease activity phase, 12 in the moderate activity phase, and 20 in the stable phase. Additionally, 20 healthy controls were included. In the validation phase, 60 cases were included, consisting of 20 patients with high disease activity of SLE, 20 patients in the moderate activity phase, 20 patients in the stable phase, and 20 healthy controls. The SLEDAI score was assessed as follows: Scores of 0-9 were classified as mildly active, 10-14 as moderately active, and ≥15 points as severely active.^12^ All SLE patients were treated for the first time in the hospital, and no glucocorticoids or immunosuppressants were used.

A total of 68 subjects were included in this study, all of whom were Han Chinese. There were no significant differences in age (*P* = .187) between the 2 groups (Table 1).

### Extraction and Identification of Exosomes

Plasma samples from both SLE patients and healthy controls were centrifuged at 500 × g for 10 minutes. The supernatant was transferred to a new tube, followed by centrifugation at 2000 × g for 30 minutes. Subsequently, the upper layer was collected and centrifuged at 10 000 × g for 30 minutes. The supernatant was filtered through an aseptic filter and centrifuged at 120 000 × g for 60 minutes. The obtained exosomes were then stored at −80°C. Transmission electron microscopy (TEM, JEOL, Japan) was employed to observe the purity of the exosome suspension and morphology. Nanosight Zetaview nanoparticle tracking analyzer (ZetaVIEW S/N 17-310, PARTICLE METRIX) was used to observe the exosome morphology and measure their particle distribution to determine the purity and concentration of exosomes. Specific exosomal surface markers (CD9 and CD63) were detected using western blotting after measuring the protein concentration of isolated exosomes using the BCA protein detection kit (ThermoFisher Scientific, USA).

### Gene Chip Detection

Total RNA was extracted from the exosome suspension and sent to Shanghai Huaying Biological Co., Ltd. for chip hybridization and data acquisition.

### Quantitative Real-Time Polymerase Chain Reaction Using an External Standard

Plasma exosomes were extracted from each SLE patient. Then, 1 mL of QIAGEN extraction reagent was added, the suspensions were fully mixed, 200 µL of chloroform was added, and they were left for 5 min at room temperature. Next, they were centrifuged at 4˚C and 12 000 rpm for 15 minutes. The supernatant was transferred to a new Eppendorf tube, and 500 μL of isopropyl alcohol was added and mixed thoroughly before centrifugation at 4˚C and 13 000 rpm for 15 minutes. Subsequently, to obtain total RNA, 80% ethanol was added to the EP tube and allowed to precipitate. cDNA was synthesized using an miRNA 1st Strand cDNA Synthesis Kit (Vazyme Biotech, Beijing, China). Ultrasensitive detection of tsRNAs was performed using the miRNA Universal Synergetic Binding Reagent (SYBR) qPCR Master Mix (Vazyme Biotech). All amplifications were performed in triplicate. mRNA expression levels were determined using the 2^−∆∆Ct^ method. The following primer sequences were used: let-7a-5p F, 5′-GGGAG AAGTCCGCTGGTGTTG-3′ and R, 5′-CTGATCTCCTTGTTCAAGTTCA-3′, miR-23a-3p F, 5′‐CCAATTGCGCCTTCAGGCTA‐3′ and R, 5′‐CGGCAGAGTCCTTACCCACA‐3′; miR-4532 F, 5′-CCCACCCCTTGCCTATAATC-3′ and R, 5′-TTCAGGGTTGCTCTG TTCAA-3′; and *U6* F, 5′- CTCGCTTCGGCAGCACA-3′ and R, 5′- AACGCTTCACGAATTTGCGT TTTC-3′.^13^

### Statistical Analysis

All analyses were conducted using SPSS version 27.0 (IBM SPSS Corp.; Armonk, NY, USA). Categorical variables are presented as frequencies and compared using the chi-square test. Data were non-normally distributed and exhibited heterogeneity of variances; they were summarized as medians and interquartile ranges. Comparisons among the 3 disease activity groups were conducted using the Kruskal–Wallis test, followed by Dunn’s multiple comparisons test where appropriate. Correlations between variables were analyzed using Spearman’s rank correlation coefficient. A two-tailed *P* value of <.05 was considered statistically significant.

## Results

### Clinical Data of Enrolled Patients

Among the 48 SLE patients, the majority of whom initially presented with facial erythema or joint pain. There were no statistically significant differences in age, sex distribution, WBC, RBC, or immunoglobulin G levels among the SLE groups (all *P* > .05). However, PLT counts were significantly lower in the high and medium activity groups compared with the low activity groups (*P* = .002). Similarly, Complement C3 and C4 levels were significantly decreased in patients with higher disease activity (*P* = .001 for both), while ESR and 24-hour urinary protein levels were significantly elevated in the high disease activity group compared to other groups (*P* = .004 and *P* = .009, respectively) (Table 1).

### Identification of Exosomes in Systemic Lupus Erythematosus Patients

**Figure 1.**
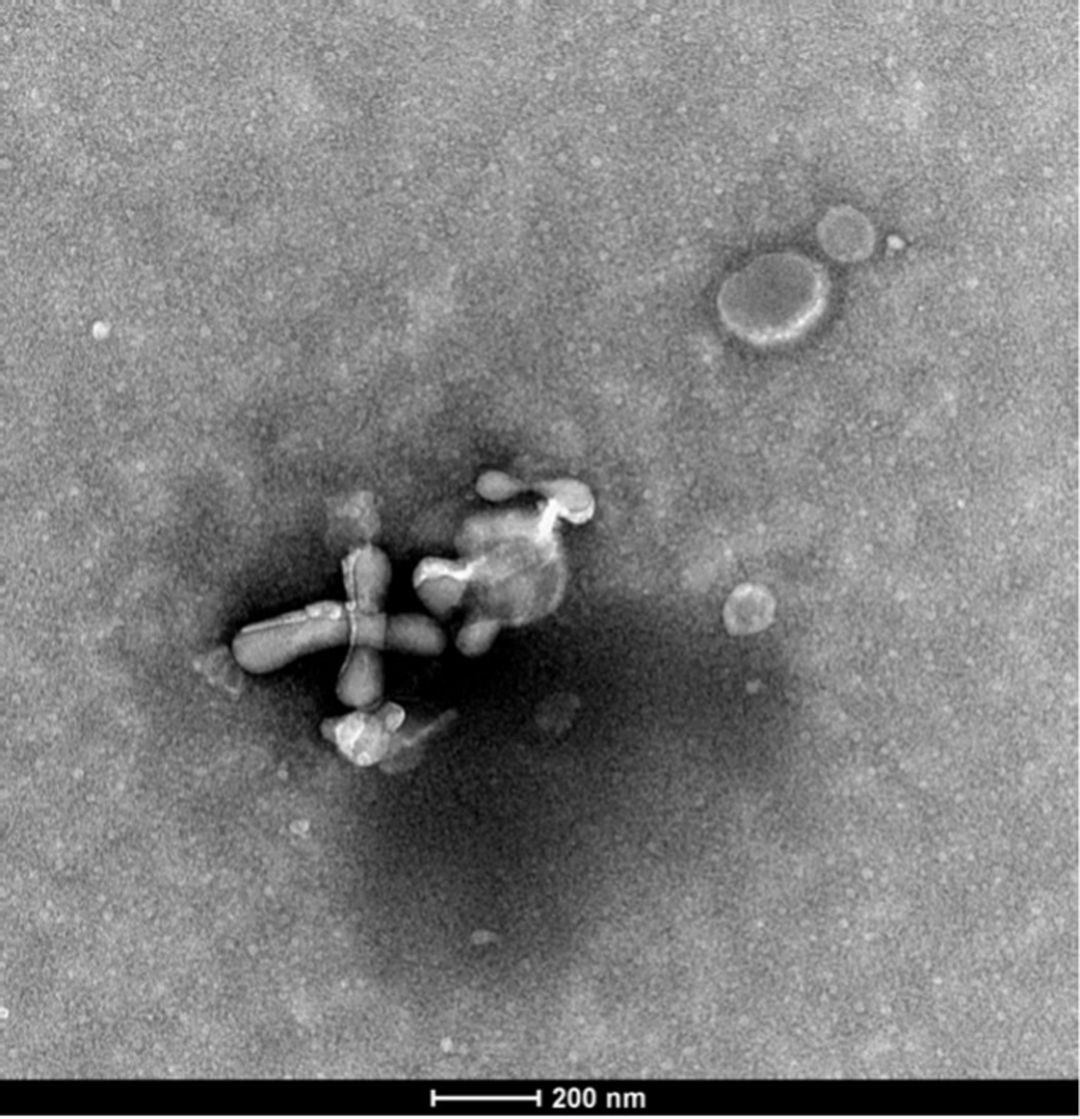
Transmission electron microscopy image of exosomes.

**Figure 2.**
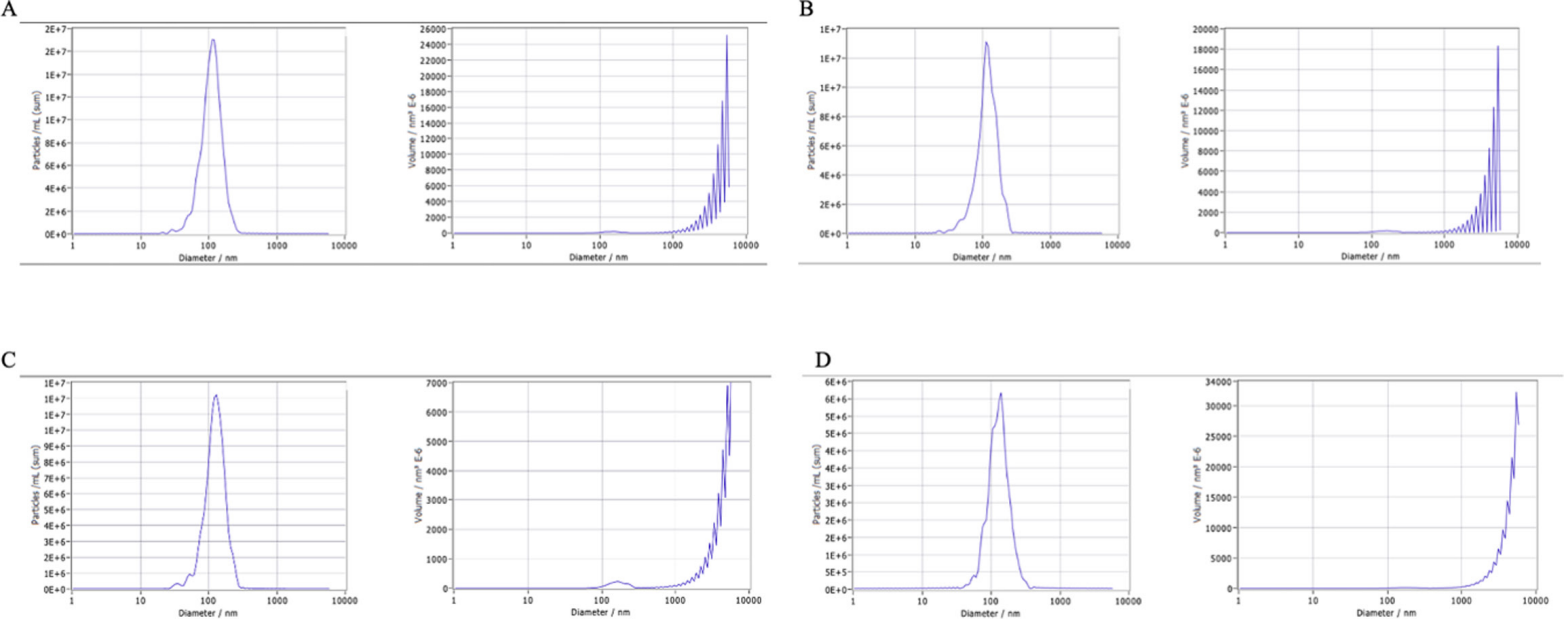
The purity and concentration of exosomes. A, Exosomes in systemic lupus erythematosus (SLE) patients with high disease activity. B, Medium disease activity of SLE patients. C, Low and medium disease activity of SLE patients. D, Normal control.

**Figure 3.**
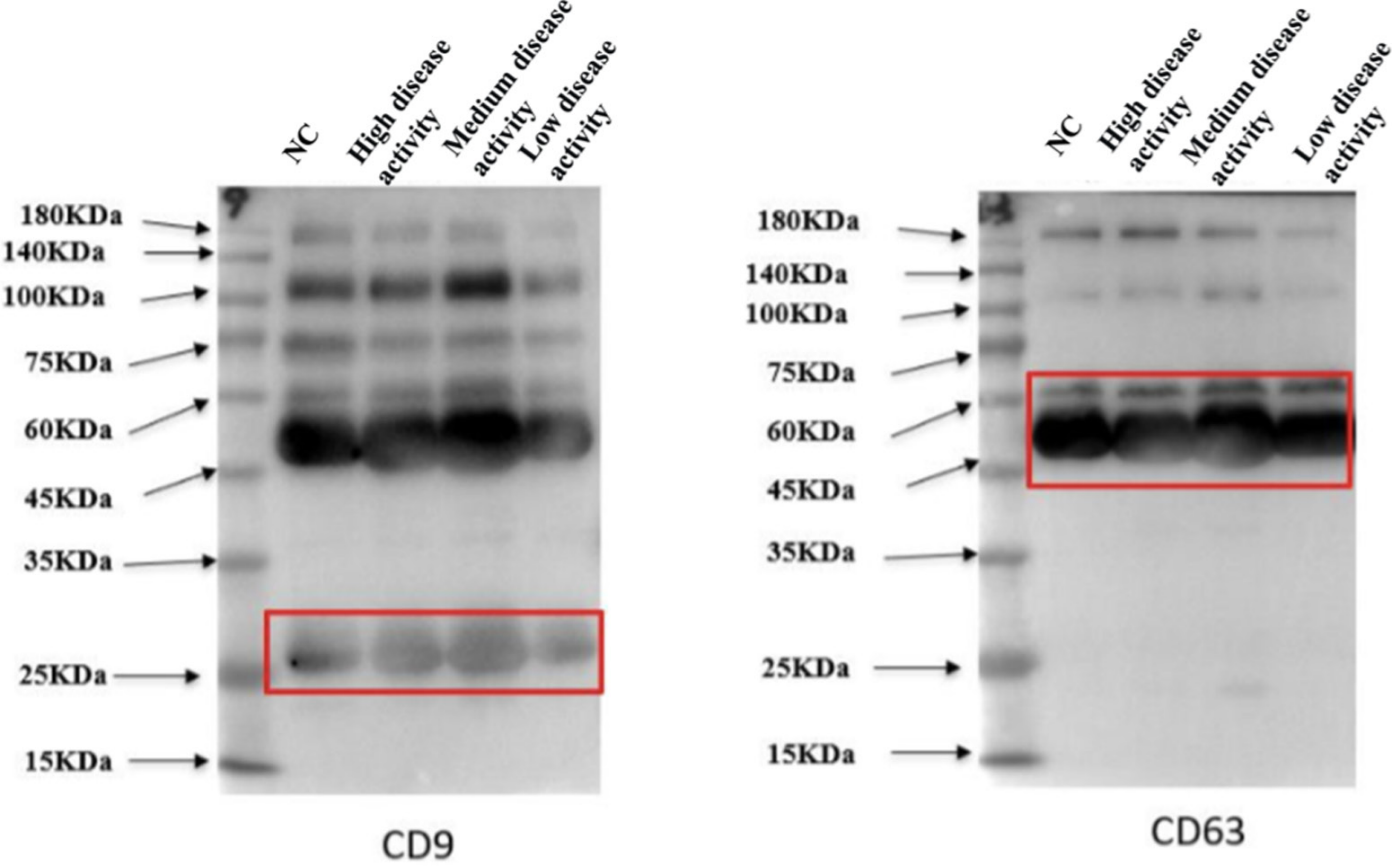
Expression of surface markers CD9 and CD63 in normal control and systemic lupus erythematosus patients.

Upon identification using TEM, the successful extraction of exosomes (Figure 1) was confirmed, which is consistent with previously described exosomal structures. All exosome samples were sent to Shanghai Huayin Biomedical Technology Co., Ltd. for nanoparticle tracking analysis to measure particle size and concentration (Figure 2). The exosome particle size in SLE patients with high disease activity was the smallest. In terms of particle concentration, the highest concentration was observed in the low and moderate disease activity groups of SLE patients, followed by the high disease activity group, with the lowest concentration found in the normal control group (Table 2). Surface markers on the extracted exosomes were also detected (Figure 3), with CD9 and CD63 expression observed in both the control and SLE patient exosomes.

### Plasma Exosomal Gene Analysis

**Figure 4.**
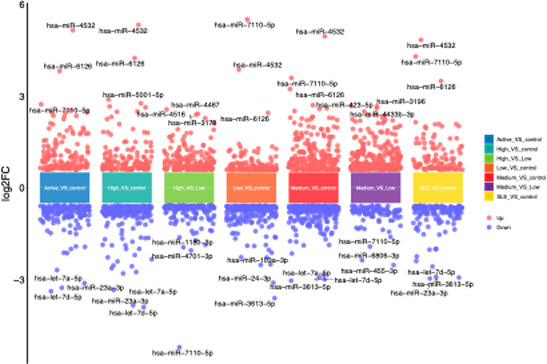
Volcano map of exosomal miRNA in systemic lupus erythematosus patients and normal control.

After performing microarray analysis on the submitted samples, library construction was completed by Huayin Biomedical Technology Co. Using the database, an overall analysis of differential genes in exosomes was conducted among 3 groups of SLE patients with varying disease activity levels and healthy controls. The comparisons included: between high and low disease activity within SLE subgroups (high vs. low, medium vs. low), between varying disease activity of SLE and healthy controls (high vs. control, medium vs. control, low vs. control), as well as between the overall SLE group and healthy controls (SLE vs. control), and between the relatively active SLE group (active, high + medium) and healthy controls (active vs. control). The results were visualized using volcano plots to represent the overall distribution of each group (Figure 4). In the plots, the x-axis represents the log2-transformed fold change of gene expression, while the y-axis represents the −log10-transformed *P*-value indicating the significance of differential expression. Red dots represent upregulated genes, and blue dots represent downregulated genes. Among the consistently differentially expressed miRNAs across multiple comparisons, let-7a-5p, miR-23a-3p, and miR-4532 were identified as top-ranking candidates due to their recurrent and statistically significant differential expression, as well as their potential biological relevance. let-7a-5p and miR-23a-3p showed a consistent downregulation pattern in patients with high disease activity compared to those with low activity or controls. In contrast, miR-4532 was significantly upregulated in patients with higher disease activity levels.

### Expression of Exosomes let-7a-5p, miR-23a-3p, and miR-4532 in Systemic Lupus Erythematosus Patients

**Figure 5.**
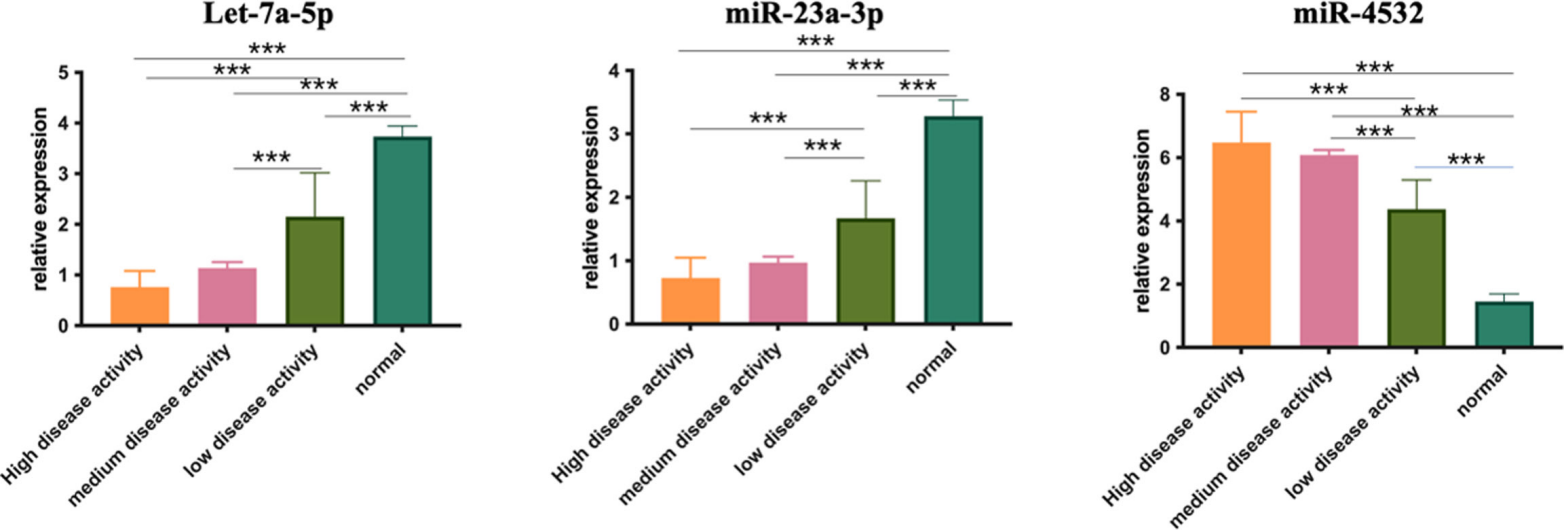
Relative expression levels of let-7a-5p, miR-23a-3p, and miR-4532 in patients with different disease activity of systemic lupus erythematosus. *P < .05.

Through the volcano map, 3 genes that were differentially expressed and appeared more frequently in different SLE disease activity groups were screened for. As shown in Figure 5, exosomal let-7a-5p and miR-23a-3p exhibited significant decreases in SLE patients at a significantly active stage compared to those at a stable stage (*P* < .0001), whereas the level of exosomal miR-4532 was found to be increased in patients with significant disease activity (*P* < .0001).

### Correlation of Exosomal miRNAs with Clinical Parameters

The correlations between serum exosomal miRNAs and various clinical parameters were further analyzed. Specifically, in the high disease activity group, let-7a-5p expression demonstrated a strong positive correlation with complement C3 (*r* = 0.692, *P* = .026) and moderate positive correlations with platelet count (*r* = 0.608, *P* = .001) and complement C4 (*r* = 0.573, *P* = .084). It also exhibited moderate negative correlations with ESR (*r* = –0.648, *P* = .043), 24-hour urinary protein (*r* = –0.406, *P* = .044), and SLEDAI score (*r* = –0.467, *P* = .026). In the medium disease activity group, let-7a-5p was moderately positively correlated with platelet count (*r* = 0.537, *P* = .015), complement C3 (*r* = 0.575, *P* = .032), and C4 (*r* = 0.548, *P* = .063). It showed moderate negative correlations with ESR (*r* = –0.671, *P* = .034), urinary protein (*r* = –0.543, *P* = .005), and SLEDAI score (*r* = –0.514, *P* = .012). In the low activity group, no statistically significant correlations were observed (all *P* > .05) (Table 3).

Regarding miR-23a-3p, in the high disease activity group, its expression showed a weak negative correlation with serum C3 (*r* = −0.476, *P* = .041). Meanwhile, it was weakly and positively correlated with 24-hour urinary protein (*r* = 0.485, *P* = .007). In the medium disease activity group, miR-23a-3p exhibited a moderate positive correlation with ESR (*r* = 0.671, *P* = .034) (Table 4).

For miR-4532, in the high disease activity group, its expression was moderately and positively correlated with ESR (*r* = 0.627, *P* = .028) and 24-hour urinary protein (*r* = 0.564, *P* = .012), while showing a weak positive correlation with the SLEDAI score (*r* = 0.493, *P* = .003). Additionally, serum C3 (*r* = −0.670, *P* = .034) and C4 (*r* = −0.671, *P* = .021) were moderately and negatively correlated with miR-4532 expression. In the medium disease activity group, miR-4532 showed a weak correlation with SLE score (*r* = 0.402, *P* = .049) (Table 5).

## Discussion

In this study, 3 exosomal miRNAs—let-7a-5p, miR-23a-3p, and miR-4532—were identified as showing differential expression across SLE disease activity states and were found to be associated with clinical markers of inflammation and renal involvement, suggesting their potential value in disease assessment and targeted therapy.

Exosomes have emerged as a promising focus in autoimmune disease research. Increasing attention has been given to their roles in immune regulation, including antigen presentation, immune activation, immunosuppression, and immune surveillance.^14-18^

In this study, exosomes from SLE patients with high disease activity exhibited the smallest particle size but the highest concentration among all groups, with statistically significant differences. Previous studies on high-grade gliomas have shown that smaller exosomes enhance tumor aggressiveness. Furthermore, the smaller the exosome size, the more readily they are absorbed by target cells, facilitating faster intercellular communication.^19^ However, further studies with larger sample sizes are needed to validate the association between exosome concentration, particle size, and SLE disease activity.

To analyze the miRNA composition of plasma exosomes in SLE patients with different disease activities and healthy controls, microarray analysis was used to identify specifically expressed miRNAs. The findings revealed 73 differentially expressed miRNAs among different disease activity groups, of which 49 were upregulated and 24 were downregulated. The investigation demonstrates the differential expression of let-7a-5p, miR-23a-3p, and miR-4532 in exosomes. These findings were further validated using PCR.

One of these differentially expressed miRNAs was let-7a-5p, which belongs to the let-7 family. Extensive research on let-7 miRNAs has demonstrated their significance. Tang et al^20^ generated induced pluripotent stem cells from renal tubular cells isolated from the urine of SLE patients and healthy controls to analyze miRNA expression profiles. Among the 223 differentially expressed miRNAs, the expression of let-7a-5p in SLE patients was significantly downregulated compared to the control group.^20^ Consistently, this study also demonstrated similar results. In this study, compared with stable SLE patients, the expression of exosomal let-7a-5p was significantly downregulated in patients with active SLE. Furthermore, the content of let-7a-5p was negatively correlated with SLEDAI score in both high and low disease activity groups. In addition, let-7a-5p was somewhat correlated with PLT and complement C3 and C4 levels in the high disease activity group. This may be due to the consumption of complement by a large number of autoantibodies via the classical pathway, leading to complement activation during the active disease phase. However, this correlation was weak during the disease stabilization phase. Moreover, let-7a-5p levels were negatively correlated with ESR and 24-hour urinary protein in patients with high disease activity. These findings suggest that plasma exosomal let-7a-5p may serve as an indicator of kidney injury, distinguishing between active and inactive stages of SLE. However, the exact mechanism remains unclear. Studies have shown that let-7a-5p forms a feedback loop with STAT3 and hnRNP-A1 to regulate PKM2 expression, thereby influencing glucose metabolism in breast cancer cells and representing a potential therapeutic target for breast cancer.^21^ Moreover, in chronic inflammatory conditions such as chronic sinusitis, let-7a-5p regulates the inflammatory response via the Ras-MAPK pathway.^22^

Similarly, miR-23a-3p has been extensively investigated in various autoimmune diseases. Studies have shown significant expression differences in 27 miRNAs, including miR-23a-3p, between RA patients and controls.^23^ In hand osteoarthritis (OA), 40 miRNAs were differentially expressed compared to healthy controls. Among them, 3 miRNAs—miR-23a-3p, miR-146a-5p, and miR-652-3p—were upregulated in hand OA patients compared to healthy controls.^24^ It was found that exosomal miR-23a-3p expression was significantly downregulated in active SLE patients compared to stable SLE patients. During high disease activity, miR-23a-3p showed a positive correlation with ESR, 24-hour urinary protein, and serum C4 levels, suggesting that miR-23a-3p levels increase with the severity of inflammation and disease activity. Moreover, miR-23a-3p was negatively correlated with C3 in the high disease activity group.

miR-4532 has received limited attention in autoimmune diseases. Overexpression of miR-4532 has been shown to significantly promote endothelial injury. However, pretreatment with a miR-4532 inhibitor or GW4869 (an exosome inhibitor) reversed this effect.^25^ Another study investigated patients with Immunoglobulin A (IgA) nephropathy and healthy controls. Urinary exosomes were isolated, and miRNA was subsequently extracted. In comparison to healthy controls, miR-4532 expression was significantly downregulated, suggesting its potential as a non-invasive biomarker for the diagnosis of IgA nephropathy.^26^ In this study, miR-4532 showed a negative correlation with complement levels (C3 and C4) in the high disease activity group. It was speculated that excessive autoantibodies in SLE may lead to increased complement consumption. Moreover, miR-4532 expression was positively correlated with ESR, 24-hour urinary protein, and SLEDAI scores, suggesting a potential role for miR-4532 in SLE progression.

This study has some limitations. First, due to the relatively short study duration, the number of plasma samples collected from SLE patients was limited. Second, Future longitudinal studies are warranted to explore dynamic changes in miRNA profiles during disease progression or in response to treatment, which may provide deeper insights into the functional roles of these miRNAs in SLE pathogenesis.

In conclusion, differentially expressed miRNAs in exosomes may be involved in regulating SLE disease severity. Notably, this research enhances the understanding of the immune mechanisms underlying SLE. Furthermore, it provides insights into the potential development of novel targeted immunotherapies via exosome-mediated mechanisms.

## Figures and Tables

**Table 1.** General Demographic and Clinical Data of Systemic Lupus Erythematosus Patients and Control Group

Basic Information / Clinical Features	SLE (N = 48)			Control (n = 20)	Z/H	*P*
	High Disease Activity (n = 16)	Medium Disease Activity (n = 12)	Low Disease Activity (n = 20)			
Age, years	32 (28, 46)	30 (22, 42)	48 (36, 55)	32 (24, 47)	4.80	.187
Sex (M/F)	0/16	0/12	1/19	0/20	2.436	.487
WBC, 10^9^/L	3.16 (1.78, 4.51)	3.98 (2.94, 4.43)	5.36 (3.24, 6.96)	/	3.68	.159
RBC, 10^12^/L	3.30 (3.05, 4.45)	3.98 (3.40, 4.38)	3.16 (1.78, 4.51)	/	3.71	.157
PLT, 10^9^/L	24 (10, 72)	100 (86.5, 122)	212 (168.25, 276.50)	/	12.37	.002*
ESR, mm/h	98 (53.50, 145)	34 (27.50, 56)	18 (9.50, 19.50)	/	11.18	.004*
24-h urinary protein, g	0.76 (0.51, 1.47)	0.47 (0.22, 1.36)	0.17 (0.07, 0.20)	/	10.21	.009*
Immunoglobulin G, g/L	20.50 (16.98, 25.15)	16.80 (16.25, 28.51)	18.60 (12.45, 20.10)	/	4.04	.102
Complement C3, g/L	0.27 (0.21, 0.43)	0.49 (0.42, 0.58)	0.78 (0.79, 0.88)	/	10.48	.001*
Complement C4, g/L	0.05 (0.04, 0.06)	0.11 (0.05, 0.14)	0.18 (0.16, 0.25)	/	13.69	.001*
dsDNA, IU/mL	>100	49.57 (45.95, 52.95)	3.39 (1.33, 5.42)	/	10.93	.001
SLEDAI	16 (15, 17)	11 (10,13)	5 (3, 7)	/	12.03	.001*

ESR, erythrocyte sedimentation rate; PLT, platelet count; RBC, red blood cells; WBC, white blood cells.

*P < .05.

**Table 2.** Nanoparticle Tracking Analysis of Exosomes in Systemic Lupus Erythematosus Patients and Normal Controls

	High Disease Activity	Medium Disease Activity	Low Disease Activity	Normal Controls	*P*
Particle size (nm)*	106.82 ± 1.47	113.3 ± 1.58	121.8 ± 0.71	124.45 ± 0.48	.001
Particle concentration (Particles/mL)*	(1.79E + 12) ±(9.62E + 10)	(4.20E + 12) ±(9.89E + 9)	(4.04E + 12) ±(3.54E + 10)	(5.72E + 11) ±(2.79E + 10)	.001

*P < .05.

**Table 3.** Correlation of Exosomal let-7a-5p with Clinical Parameters

Variables	High Disease Activity		Medium Disease Activity		Low Disease Activity	
	*r*	*P*	*r*	*P*	*r*	*P*
PLT, 10^9^/L	0.608*	.001*	0.537*	.015*	0.241	.141
ESR, mm/h	−0.648*	.043*	−0.671*	.034*	0.314	.060
24-h urinary protein, g	−0.406*	.044*	−0.543*	.005*	−0.396	.206
Complement C3, g/L	0.692*	.026*	0.575*	.032*	0.401*	.251
Complement C4, g/L	0.573*	.084	0.548*	.063	0.414*	.234
SLEDAI	−0.467*	.026*	−0.514*	.012*	−0.255	.056

ESR, erythrocyte sedimentation rate; PLT, platelet count.

*P < .05.

**Table 4.** Correlation of Exosomal miR-23a-3p with Clinical Parameters

Variables	High Disease Activity		Medium Disease Activity		Low Disease Activity	
	*r*	*P*	*r*	*P*	*r*	*P*
PLT, 10^9^/L	0.333	.049*	0.604*	.064	0.235	.094
ESR, mm/h	0.379	.008*	0.671*	.034*	−0.316	.058
24-h urinary protein, g	0.485*	.007*	0.372	.047*	−0.203	.055
Complement C3, g/L	−0.476*	.041*	0.354	.316	0.389	.419
Complement C4, g/L	0.531*	.051	0.214	.378	0.221	.122
SLEDAI	0.367	.006*	0.252	.058	−0.292	.076

ESR, erythrocyte sedimentation rate; PLT, platelet count.

*P < .05.

**Table 5.** Correlation of Exosomal miR-4532 with Clinical Parameters

Variables	High Disease Activity		Medium Disease Activity		Low Disease Activity	
	*r*	*P*	*r*	*P*	*r*	*P*
PLT, 10^9^/L	−0.252	.102	−0.591*	.195	−0.231	.212
ESR, mm/h	0.627*	.028*	0.414*	.296	−0.292	.149
24h urinary protein, g	0.564*	.012*	0.272	.059	0.384	.051
Complement C3, g/L	−0.670*	.034*	−0.346	.073	0.313	.177
Complement C4, g/L	−0.671*	.021*	−0.372	.044*	0.242	.197
SLEDAI	0.493*	.003*	0.402*	.049*	0.174	.096

ESR, erythrocyte sedimentation rate; PLT, platelet count.

*P < .05.

## Data Availability

The data that support the findings of this study are available on request from the corresponding author.
